# Exploring the Solid-State Landscape of Carbamazepine during Dehydration: A Low Frequency Raman Spectroscopy Perspective

**DOI:** 10.3390/pharmaceutics15051526

**Published:** 2023-05-18

**Authors:** Peter III J. G. Remoto, Kārlis Bērziņš, Sara J. Fraser-Miller, Timothy M. Korter, Thomas Rades, Jukka Rantanen, Keith C. Gordon

**Affiliations:** 1 The Dodd-Walls Centre for Photonic and Quantum Technologies, Department of Chemistry, University of Otago, Dunedin 9016, New Zealand; rempe782@student.otago.ac.nz (P.I.J.G.R.); karlis.berzins@sund.ku.dk (K.B.); 2 Department of Chemistry, Center for Science and Technology, Syracuse University, Syracuse, NY 13244, USA; tmkorter@syr.edu; 3 Department of Pharmacy, Faculty of Health and Medical Sciences, University of Copenhagen, 2100 Copenhagen, Denmark; thomas.rades@sund.ku.dk (T.R.); jukka.rantanen@sund.ku.dk (J.R.)

**Keywords:** carbamazepine, low-frequency Raman spectroscopy, THz Raman spectroscopy, dehydration, solid-state, polymorphism

## Abstract

The solid-state landscape of carbamazepine during its dehydration was explored using Raman spectroscopy in the low- (−300 to −15, 15 to 300) and mid- (300 to 1800 cm^−1^) frequency spectral regions. Carbamazepine dihydrate and forms I, III, and IV were also characterized using density functional theory with periodic boundary conditions and showed good agreement with experimental Raman spectra with mean average deviations less than 10 cm^−1^. The dehydration of carbamazepine dihydrate was examined under different temperatures (40, 45, 50, 55, and 60 °C). Principal component analysis and multivariate curve resolution were used to explore the transformation pathways of different solid-state forms during the dehydration of carbamazepine dihydrate. The low-frequency Raman domain was able to detect the rapid growth and subsequent decline of carbamazepine form IV, which was not as effectively observed by mid-frequency Raman spectroscopy. These results showcased the potential benefits of low-frequency Raman spectroscopy for pharmaceutical process monitoring and control.

## 1. Introduction

Pharmaceutical products must be carefully developed and formulated to ensure that they are safe and of the desired quality for general use [1]. The majority (85%) of active pharmaceutical ingredients (APIs) exist as polymorphs (differently arranged molecules in a crystalline lattice) and solvates or hydrates (incorporation of solvent or water molecules in the crystal lattice). Therefore, the detection and characterization of solid-state transformations throughout the manufacturing process is important to ensure consistency and efficacy, as different solid forms will affect the performance of a given pharmaceutical [2,3]. Raman spectroscopy is a well-known and established process analytical technology (PAT) tool, and its rapid, non-destructive characteristics have been widely exploited [4,5]. This technique probes the molecular vibrations of chemical structures, providing a “molecular fingerprint” which can be used for their characterization. In the pharmaceutical setting, Raman spectroscopy has shown a variety of applications, including solid-state characterization [6,7,8], monitoring of dissolution and solubilization processes [9], and in-line quantification [10,11]. Low-frequency Raman (LFR) spectroscopy, also known as THz or low-wavenumber Raman spectroscopy, is a burgeoning technique which probes the low-energy lattice vibrational motions (also referred to as intermolecular vibrational or phonon modes). This technique is very useful for gaining more information about the internal structure of a sample, such as phase transformations of a solid [12]. With LFR spectroscopy (<300 cm^−1^), amorphous (non-crystalline) solids manifest a broad halo-like shape (broad vibrational density of states), whilst crystalline solids tend to exhibit sharp spectral features or so-called phonon modes.

Water can interact with solids in a number of ways, including physisorption (surface binding), absorption, chemical addition, fluid inclusion, and hydrate formation [13,14]. The ability of a substance to non-covalently absorb moisture from its surroundings is referred to as hygroscopicity. As a result, hygroscopic materials are particularly susceptible to environments with higher relative humidity. One class of solid-state forms, known as hydrates, incorporates water molecules within the parent crystalline structure. Van der Waals forces and hydrogen bonds are typically involved in the interaction between the parent and water molecules. Hydrates can be divided into the following types: (1) non-stoichiometric hydrates, which incorporate disordered water molecules within the crystalline structure as a function of environmental conditions such as relative humidity (RH) and temperature; and (2) stoichiometric hydrates, which contain a constant ratio of water molecules to parent molecules in a well-organized structure [15]. Additionally, hydrates can be classified into different semi-empirical classes, such as ‘isolated site’ and ‘channel-type’ hydrates [16]. Differences in the molecular packing of hydrates can affect dehydration processes. In this study, we focus on a crystal structure that incorporates water molecules into channels within the structural lattice arranged in a column-like array [17].

Previous work has described the use of Raman spectroscopy in the low- and mid-frequency regions for monitoring the solid-state transformation of piroxicam monohydrate, theophylline monohydrate, and nitrofurantoin monohydrate II [7,18]. It was found that mid-frequency Raman (MFR) spectroscopy (300 to 1800 cm^−1^) detected the onset of nitrofurantoin monohydrate II dehydration faster than LFR spectroscopy. It was hypothesized that this was because MFR detected the changes in hydrogen bonding between the water and nitrofurantoin molecules. In the cases of piroxicam monohydrate and theophylline monohydrate, which are channel hydrates, LFR detected changes before the MFR. This was attributed to the sensitivity of low-frequency modes to the mobility of the water molecules in the channels. These results have implied that LFR and MFR spectroscopies offer unique advantages to monitor different aspects of structural rearrangements during the dehydration processes.

**Figure 1:**
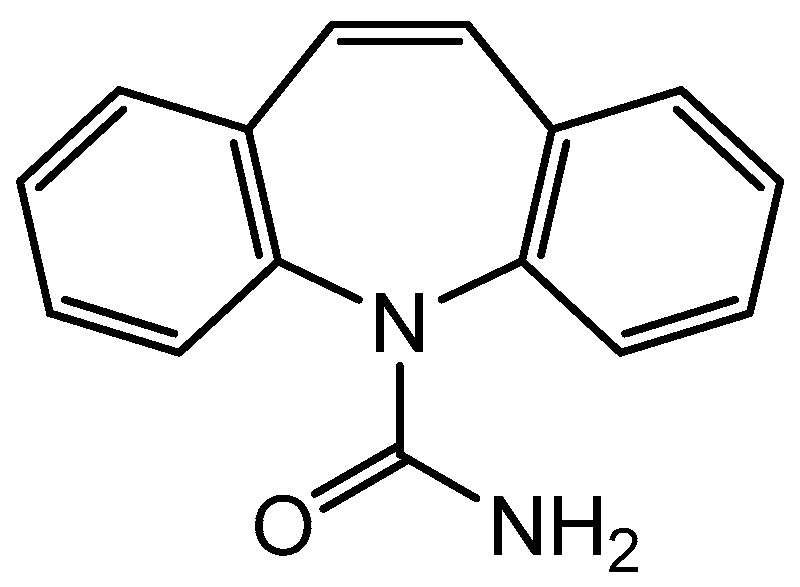
Molecular structure of carbamazepine.

Here, we extend our studies of API hydrates and their dehydration behavior by exploring carbamazepine and its solid-state transformations during the dehydration of its dihydrate form. Carbamazepine (CBZ, Figure 1) is a pharmaceutical widely used as an anticonvulsant to treat epilepsy and neuropathic pain [19]. Carbamazepine has an interesting solid-state landscape as it has four known dimeric anhydrous forms (Forms I, II, III, IV) [20,21,22], a recently discovered catemeric anhydrous form (Form V), and only one hydrated form—a channel type dihydrate [23,24]. The dehydration of carbamazepine has been previously investigated in detail [24,25,26]. Kachrimanis and Griesser investigated the dehydration of CBZ-DH single crystals using thermogravimetric analysis (TGA) and optical microscopy [24]. Their findings showed that the resulting product of the dehydration of CBZ varied depending on the environmental conditions (relative humidity (RH) and temperature). For example, the dehydration of CBZ-DH at room temperature (~20 °C) produced amorphous CBZ at 0% RH, CBZ-I was produced at ~10% RH, and a mixture of CBZ-I and CBZ-III at higher RH. Furthermore, CBZ-DH dehydrated between 50 and 100 °C produced a mixture of semi-crystalline CBZ-I and CBZ-IV. Li et al. used TGA and powder X-ray diffraction (PXRD) to characterize amorphous CBZ after CBD-DH dehydration under specific conditions (nitrogen gas 200 mL min^−1^, 45 °C, 20 min) [25]. Kogermann et al. used MFR and PXRD to monitor the dehydration of CBZ-DH. Their findings showed that the dehydration of CBZ-DH produced amorphous CBZ, CBZ-I, and CBZ-III, of which the quantities varied with temperature.

In this study, we use LFR and MFR to characterize the differing forms of CBZ and examine the solid-state changes as the dihydrate form is heated. We also provide density functional theory calculations with periodic boundary conditions to aid in our low-frequency Raman normal mode assignments and identification of the differing forms of CBZ observed.

## 2. Materials and Methods

Commercial carbamazepine (Form III, 236.27 g mol^−1^, 98%, HPLC) was purchased from AK Scientific, Inc. (Union City, CA, USA). Hydroxypropyl cellulose (806.948 gmol^−1^, Mw = 60,000) was purchased from Sigma-Aldrich (Taufkirchen, Germany). These materials were stored at 20 °C and 48% relative humidity in a dark location. 

### 2.1. Preparation of Carbamazepine Solid-State Forms

The polymorphs and hydrate forms of carbamazepine were prepared using methods described in the literature [20,21,25]. 

It was not possible to synthesize CBZ form II (CBZ-II) using the methods described in the literature [8,20]. Our attempts to isolate the product resulted in CBZ-DH.

### 2.2. Raman Measurements 

Raman spectra were measured using an in-house built system that has been described previously [18]. CBZ-DH, CBZ-I, CBZ-III, CBZ-I, and CBZ-IV samples were placed on a quartz sample holder secured by an LNP95 liquid nitrogen cooler controlled THMS600 variable temperature stage (Linkam Scientific Instruments Ltd., Salfords, UK). This stage was purged with nitrogen (100 mL min^−1^) at 40 °C prior to any Raman measurements. Reference Raman measurements were performed at −190 °C and 20 °C. Each spectrum consisted of 300 co-added scans with 1 s integration time.

For the dehydration measurements, CBZ-DH (approximately 15 mg) was packed in an aluminum pan (TA Instruments, New Castle, DE, USA). A THMS600 variable temperature stage was heated from 20 °C to the target dehydration temperature of 40, 45, 50, 55, and 60 °C, and triplicate measurements of CBZ-DH samples were carried out. These selected temperatures were well below the thermal decomposition of carbamazepine (204–325 °C) to ensure that molecular degradation was avoided [27]. For each dehydration measurement, the sample was heated to the dehydration temperature at a rate of 10 °C min^−1^. The dehydration was then monitored for 90 min at the set temperature and then cooled at 10 °C min^−1^ to 20 °C. For the kinetic analysis, only the isothermal data were utilized. The dehydration temperatures ranged from above to below the glass transition temperature of CBZ; *T_g_* = 56 °C [24,25]. Each spectrum consisted of 10 scans with a 10 ms acquisition time (with CCD readout, this equates to 0.34 s). 

### 2.3. Spectral Preprocessing and Multivariate Analysis 

The Raman spectra were converted from .spe to .spc file format using SpectraGryph 1.2.15 software [28]. Spectroscopic data collected during the dehydration experiments were averaged for every 10 spectra, which represents a time period of 3.4 s. Spectra were corrected by standard normal variate (SNV) scaling followed by baseline correction. These data were analyzed via principal component analysis (PCA) using The Unscrambler X 10.5 (CAMO, Nedre Vollgate, Norway) software. Multivariate curve resolution was implemented using MATLAB (MCR-ALS Toolbox 2.0) [29].

For PCA, all temperature and replicate data were analyzed together in the low-, mid-, and combined-frequency spectral windows with 20-segment random cross-validation and the NIPALS algorithm with a maximum of 10,000 iterations. MCR was performed without initial guess spectra and with closure, non-negative spectra (fast nonnegative least squares algorithm), and non-negative concentration constraints. The analysis was constrained to four pure components as this provided the best match for the pure component curves against known polymorphs of CBZ. Using five or three components distorted the curves, resulting in a poorer match with the reference spectra. The maximum ALS iterations was set to 5000 and criterion was set to 0.05. 

### 2.4. Computational Details

Density functional theory (DFT) with periodic boundary conditions was used to perform theoretical calculations on the low-frequency Raman spectra of solid-state forms of carbamazepine (CBZ-I, CBZ-II, CBZ-III, CBZ-IV, CBZ-DH). These calculations were completed using the CRYSTAL17 software [30], where a generalized gradient approximation Perdew–Burke–Ernzerhof (PBE) [31] function with van der Waals interactions, treated according to the Grimme D3 [32] method, was used. All of the atoms were described with Ahlrichs’ VTZ basis set with added polarization functions [33,34]. This method was adapted from a previously developed generalized methodology for the vibrational analysis of small pharmaceuticals [12]. The coupled-perturbed Hartree–Fock/Kohn-Sham (CPHF/CPKS) approach [35] and the Anderson convergence accelerator [36] were used to analytically calculate the dielectric tensor and Raman intensities. Energy convergence criteria for the geometry optimization and vibrational calculations were set to ΔE ≤ 10^−8^ and 10^−10^ Hartree, respectively. The crystal structures of the CBZ-I, CBZ-II, CBZ-III, CBZ-IV, and CBZ-DH solid-state forms were fully optimized with no implied restrictions on atom positions or lattice parameters. Computational resources from the New Zealand eScience Infrastructure (NeSI) were used for these computationally expensive calculations. Theoretical vibrational modes were visualized using MOLDRAW [37]. Initial guesses for the geometry optimization of the crystal structures of CBZ-I (CSD ref: CBMZPN11) [20], CBZ-II (CSD ref: CBMZPN03) [38], CBZ-III (CSD ref: CBMZPN10) [39], CBZ-IV (CSD ref: CBMZPN12) [21], and CBZ-DH (CSD ref: FEFNOT03) [40] were taken from the Cambridge Crystallographic Data Centre (CCDC).

## 3. Results and Discussion

### 3.1. Structural Analysis 

Carbamazepine (CBZ) has five known anhydrous forms, with four being dimeric (Forms I to IV) and one form being catemeric (Form V) [20]. CBZ has one hydrate form—carbamazepine dihydrate (CBZ-DH) [24]. The nomenclature and labeling of the anhydrous CBZ solid-state forms has been inconsistent within the literature; therefore, in this study, we follow the nomenclature used by Grzesiak et al., where anhydrous CBZ forms are labelled according to their unique crystal systems as follows: triclinic is CBZ form I (CBZ-I), trigonal is CBZ form II (CBZ-II), P-monoclinic is CBZ form III (CBZ-III), and C-monoclinic is CBZ form IV (CBZ-IV) [20]. The hydrogen bonding patterns of CBZ-DH, CBZ-I, CBZ-II, CBZ-III, CBZ-IV, and CBZ-V are presented in Figure S1. 

All dimeric CBZ polymorphs displayed N-H···O and O-H···N interactions formed by the amido groups of each CBZ molecule, resulting in $R_{2}^{2}\left(8\right)$ CBZ dimers [40]. The crystal structure of carbamazepine dihydrate (CBZ-DH) exists within the monoclinic space group P2_1/c_ and belongs to the channel-type hydrate subclass. Water molecules in this system do not disrupt the $R_{2}^{2}\left(8\right)$ CBZ dimers, to which N-H···O and O-H···O interactions between water molecules and amido groups of CBZ molecules, and O-H···N between water molecules, results in $C_{3}^{2}\left(8\right)$ and $R_{8}^{4}\left(16\right)$ hydrogen bonding interactions. CBZ-DH layers are formed by water molecules displaying $C_{3}^{2}\left(8\right)$ interactions.

Different π–π interactions between neighboring CBZ molecules and varying intermolecular interactions linking each CBZ molecule give rise to distinct packing arrangements in CBZ-I, CBZ-II, CBZ-III, and CBZ-IV. CBZ-I and CBZ-II both exhibit parallel displaced π–π interactions between aromatic rings of neighboring CBZ molecules across layers of their respective lattice structures [22]. CBZ-III exhibits face-to-face π–π interactions between the aromatic rings of adjacent CBZ molecules. CBZ-IV exhibits T-shaped π–π interactions between the aromatic rings of adjacent CBZ molecules [22].

CBZ-III crystals exist within the monoclinic space group P2_1/n_ [39]. It is interesting to note that CBZ Forms I and III have an enantiotropic relationship, meaning that a reversible transformation between polymorphs can be observed with changes in temperature or pressure. CBZ-III converts to Form I at ~200 °C [27]. It has been observed that, for temperatures above 70 °C, CBZ-I is the thermodynamically stable polymorph, whereas for temperatures below 70 °C, CBZ-III is the thermodynamically stable polymorph [27,38]. CBZ-IV crystals exist in the monoclinic space group C_2/c_. The structural lattice of CBZ-IV is composed of $R_{2}^{2}\left(8\right)$ dimeric CBZ linked with C(7) interactions between azepine C-H and C=O acceptor and C(8) interactions between the aryl C-H donors and nearby C=O acceptor. CBZ-II exists in the trigonal space group $R\overset{-}{3}$ [19]. CBZ-II crystals are composed of $R_{2}^{2}\left(8\right)$ CBZ dimers linked with C(6) interactions between nearby aryl C-H and C=O acceptors. CBZ-II and CBZ-IV are the least stable at room temperature due to their packing arrangements [41]. 

**Figure 2:**
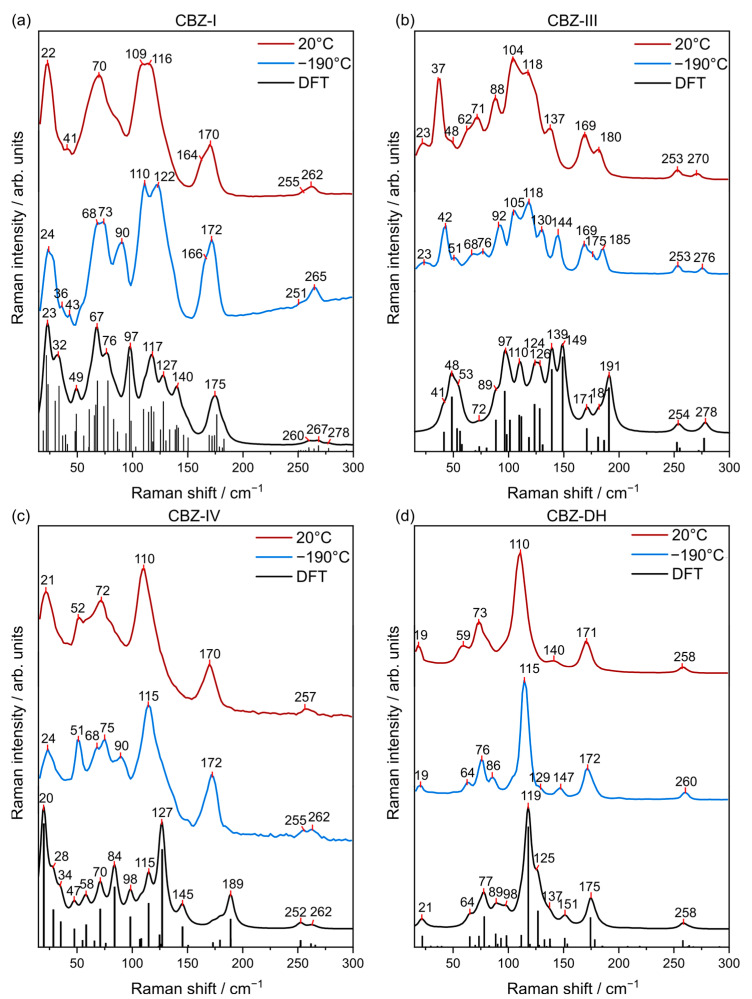
Low-frequency Raman (LFR) spectra of (**a**) CBZ-I, (**b**) CBZ-III, (**c**) CBZ-IV, and (**d**) CBZ-DH collected at 200 °C (red) and −190 °C (blue) in comparison to their DFT calculations (black).

MFR spectra of carbamazepine have been modeled using the density functional theory (B3LYP functional and 6-311++G** basis set) described by Czernicki et al. and Strachan et al. by modeling carbamazepine as a single molecule as well as a dimer [41,42]. Theoretical calculations of low-frequency Raman spectra (DFT calculations with periodic boundary conditions) of CBZ-DH, CBZ-I, CBZ-II, CBZ-III, and CBZ-IV have not been previously published in the literature. However, a number of studies have tackled the modeling of the THz absorption spectrum of CBZ polymorphs [22,43]. Initial characterization of prepared solid-state forms of CBZ can be validated using the Raman spectra of CBZ-I, CBZ-II, CBZ-III, CBZ-IV, and CBZ-DH previously seen in literature, in the low- and mid-frequency region, where possible [8,26,42,44,45,46,47]. DFT calculations with periodic boundary conditions were used to predict the Raman spectra for the various forms in the low- and mid-frequency regions (Figure 2). This provides information regarding the varying inter- and intramolecular interactions of the different carbamazepine solid-state forms. Understanding these interactions is important because it provides insight on the behavior of carbamazepine molecules in the solid-state lattice, for example, in the context of solid-state transformations. As established by previous studies, a mean absolute deviation (MAD) of less than 10 cm^−1^ was set as the criterion for satisfactory agreement between theoretical and experimental spectra [48,49,50]. So, medium to strong peaks of theoretical Raman spectra of CBZ-I (MAD = 7.8 cm^−1^), CBZ-III (MAD = 6.7 cm^−1^), CBZ-IV (MAD = 6.3 cm^−1^), and CBZ-DH (MAD = 5.4 cm^−1^) displayed good correlation with their corresponding experimental Raman spectra. Therefore, distinct peaks arising from different solid-state forms can be assigned to specific vibrational modes and help to elucidate the behavior of the solid-state form of interest [41,42].

Each CBZ solid-state form exhibited characteristic signals in the LFR spectra that clearly distinguishes them from each other. Hence, the LFR spectra of CBZ solid-state forms highlighted that, despite their identical molecular compositons and π–π interactions between neighboring CBZ molecules, they have distinct packing moeties. CBZ-DH had characteristic signals at 19 and 115 cm^−1^; CBZ-I at 24, 68, 73, 110, 122, and 267 cm^−1^; CBZ-III at 48, 97, 110, 124, 139, and 149 cm^−1^; and CBZ-IV at 20, 58, 70, 84, 127, 189, 252, and 262 cm^−1^ [41,45]. The most pronounced LFR signals of CBZ-DH, CBZ-I, CBZ-II, CBZ-III, and CBZ-IV are summarized in Tables S1–S5. The experimental LFR spectra of CBZ-I, CBZ-III, CBZ-IV, and CBZ-DH (Figure 2) were consistent with their corresponding Raman spectra found in previous studies by Larkin et al., Inoue et al., and Guinet et al. [8,46,51]. Translational and torsional modes were observed in the LFR normal modes of CBZ solid-state forms. Normal modes of CBZ-I, CBZ-III, CBZ-IV, and CBZ-DH of select frequencies are vizualized in Figures S3–S6. It is interesting to note that CBZ-I and CBZ-IV share similarities in terms of their LFR spectral band patterns at 20 °C. The LFR spectra of the CBZ solid-state forms show clear differences in contrast to the subtle differences observed using the mid-frequency Raman (MFR) region (Figure S7). In addition, the relatively large peak intensities observed in the LFR spectral region were attributed to a large change in the polarizability of solid-state phonon modes, which could originate from the backbonding between primary amide and aryl groups [8]. Nevertheless, each CBZ solid-state form has distinct signature bands and shows different relative intensities for peaks in the MFR region. For example, this can be readily seen in Figure S8 for the similar peaks of CBZ-DH (1570, 1604, and 1629 cm^−1^), CBZ-I (1574, 1600, and 1624 cm^−1^), CBZ-III (1568, 1603, 1626 cm^−1^), and CBZ-IV (1565, 1600, 1625 cm^−1^).

### 3.2. Dehydration Studies 

**Figure 3:**
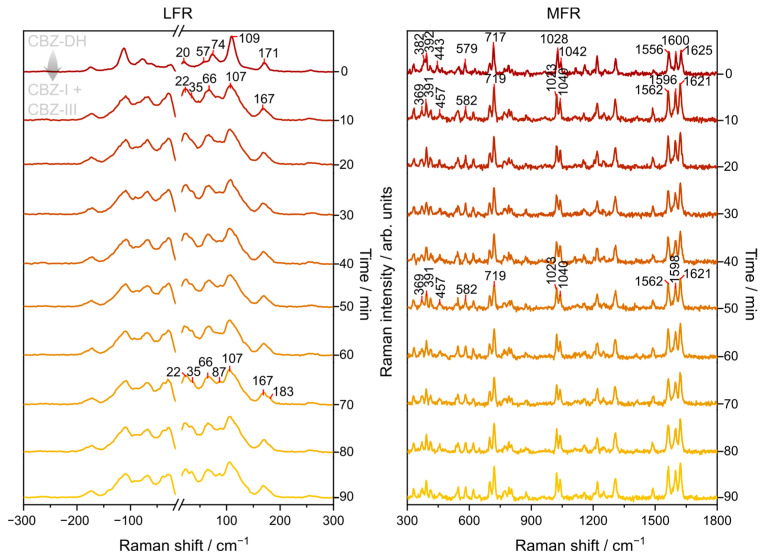
Representative LFR and MFR spectra monitoring the isothermal (at 55 °C) dehydration of CBZ-DH for 90 min.

The Raman spectra of CBZ-DH showed noticeable variations in the low- and mid-frequency regions during its isothermal dehydration, as shown in Figure 3 for the dehydration of CBZ-DH at 55 °C. There was no evidence of molecular degradation of carbamazepine molecules in this study. In the LFR region, peaks characteristic of those for CBZ-DH (20, 74, 109, and 171 cm^−1^) were replaced by signals characteristic of those for CBZ-I (22, 66, 107, and 167 cm^−1^) after 10 min. Subsequently, the growth of peaks characteristic of those for CBZ-III (35, 66, 87, 107, 183 cm^−1^) were observed and became more pronounced with time. Visual inspection of the LFR spectra indicated the transformation of CBZ-DH to a mixture of CBZ-I and CBZ-III. It is interesting to note that CBZ-I is the most thermally stable solid-state form for temperatures above 70 °C, while CBZ-III is the most thermally stable solid-state form for temperatures below 70 °C. On the other hand, the MFR region showed peaks characteristic of those for CBZ-DH (382, 392, 443, 579, 717, 1028, 1042, 1556, 1600, and 1625 cm^−1^) that were replaced by peaks at 369, 391, 457, 582, 719, 1023, 1040, 1562, 1596, 1621 cm^−1^, which indicated the formation of CBZ-I or CBZ-III. As the heating continued, the peaks at 1596 cm^−1^ blueshifted to 1598 cm^−1^, which indicated a change in solid-state form (which can allude to a progressive transformation of CBZ-I to CBZ-III). According to the MFR DFT calculations, at 1595 cm^−1^, CBZ-I displays C=C stretching, C-O stretching from its amide group, and C-N scissoring from its azepine group. At 1600 cm^−1^, CBZ-III displays C=C stretching, C-N stretching from its amide group, and C-N rocking from its azepine group. At 1600 cm^−1^, CBZ-DH exhibits C=C stretching, C-N scissoring from its azepine group, N-H bending and C-O stretching from its amide group, and O-H bending from water molecules.

Previous studies have suggested that the dehydration pathway of CBZ-DH is strongly dependent on the relative humidity (RH) and temperature of the surrounding environment with regard to the observed solid-state form of CBZ (amorphous or Forms I or III) [45,52]. Although conducted on CBZ-DH single crystals, the dehydration mechanisms of CBZ-DH have previously been described as consisting of two overlapping fast and slow processes [24]. The fast process is assumed to involve the formation of amorphous CBZ due to the dehydration of the water molecules (which were bound to the C=O group). The other dehydration process involved the following: (a) a slow formation of CBZ-IV when the dehydration temperature is lower than 50 °C; (b) a rapid formation of CBZ-I when the dehydration temperature is greater than 50 °C. This dehydration process is slower because the water molecules are more strongly bound to the N-H group. Furthermore, another CBZ-DH dehydration study found that CBZ-DH dehydrated to CBZ-I, CBZ-III, and a transient amorphous CBZ form that converts to CBZ-IV [24]. LFR spectroscopy has been used to monitor the enantiotropic relationship between CBZ-III and CBZ-I. It was found that heating CBZ-III to 170 °C increased characteristic peaks from CBZ-I, while CBZ-III peaks diminished and subsequent cooling to ambient temperatures recovered CBZ-III [51]. Overall, previous studies have indicated that there are possibly a number of solid-state forms present during the dehydration process of CBZ-DH [24,25,26,51,52]. In light of this, principal component analysis (PCA) and multivariate curve resolution (MCR) were used to detect the potential presence and change in abundance of the different solid-state forms of CBZ during dehydration. The efficacy in the detection of these forms was also compared between LFR and MFR spectroscopy. In our study, bulk powdered CBZ-DH samples were examined instead of single crystals; thus, these experiments are less sensitive to localized transformations within micron-sized domains.

### 3.3. Principal Component Analysis (PCA) 

Principal component analysis (PCA) is an unsupervised dimensionality reduction method used to visualize the variance within a dataset through generating a set of linear combinations of orthogonal variables. PCA was first used as an exploratory chemometric tool to investigate the datasets collected during the CBZ-DH dehydration process at different temperatures. 

**Figure 4:**
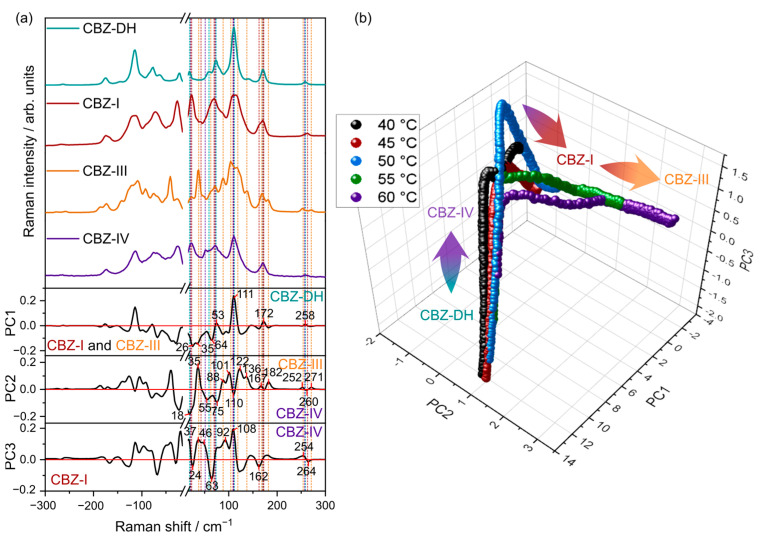
PCA loadings (**a**) and scores (**b**) for data collected from all temperatures and the low frequency Raman spectral region. For ease of visualization, the mean scores value at each time point for each replicate run is shown.

PCA loadings and scores of the low-frequency spectral region are shown in Figure 4. The score plot shows sample trajectories with multiple turning points in the data over time. The exact distance along the path varied with temperature. When inspecting the associated loadings for the first three principal components (PCs), these turning points suggest places in PC space with a higher relative abundance of a particular polymorph, with the approximate trajectory of CBZ-DH to CBZ-IV to CBZ-I to CBZ-III. This is consistent with published data, which demonstrate the growth of multiple CBZ solid-state forms during the dehydration of CBZ-DH [24,25,26,45,52]. The results from similar analyses carried out on the MFR and the combined (LFR+MFR) regions are given in Figures S8 and S9, respectively. The PC scores for both MFR and the combined regions show multiple turning points that suggest the growth of multiple CBZ polymorphs. The first, third, and fourth PC loadings of MFR data and the first three PC loadings of combined data domains suggested the appearance of CBZ-I, CBZ-III, and CBZ-IV during the dehydration of CBZ-DH.

However, the results from PCA did not provide evidence for the formation of amorphous CBZ during the isothermal dehydration of CBZ-DH. Therefore, the dataset was further evaluated by using MCR to direct the fitting algorithm to test for the presence of amorphous CBZ.

### 3.4. Multivariate Curve Resolution (MCR) 

**Figure 5:**
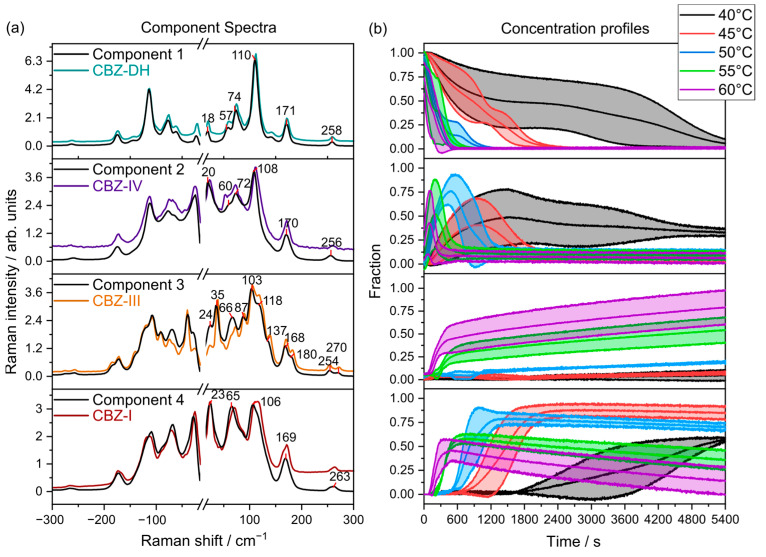
(**a**) LFR component spectra overlaid with their corresponding reference spectra (colored lines) collected at 20 °C (CBZ-DH, CBZ-IV, CBZ-III, and CBZ-I). (**b**) Mean component concentrations between triplicate runs with standard deviations (shaded, n=3).

**Figure 6:**
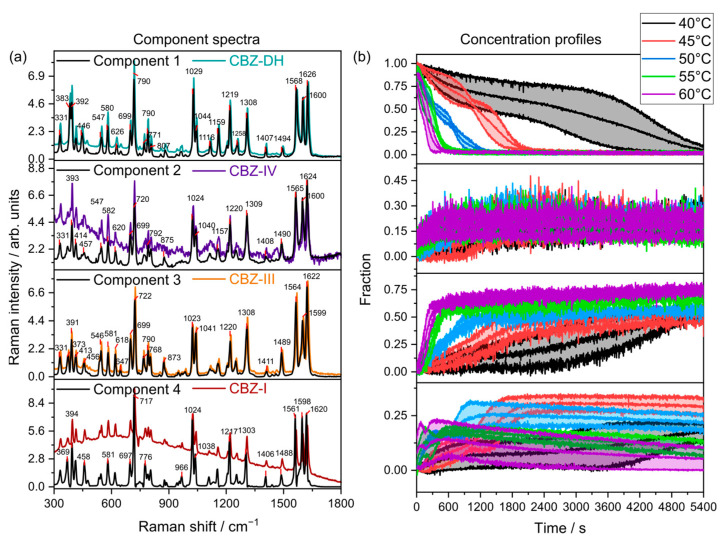
(**a**) MFR component spectra (n=3, solid lines) overlaid with their corresponding reference spectra (colored lines) collected at 20 °C (CBZ-DH, CBZ-IV, CBZ-III, and CBZ-I). (**b**) Mean component concentrations between triplicate runs with standard deviations (shaded, n=3).

**Figure 7:**
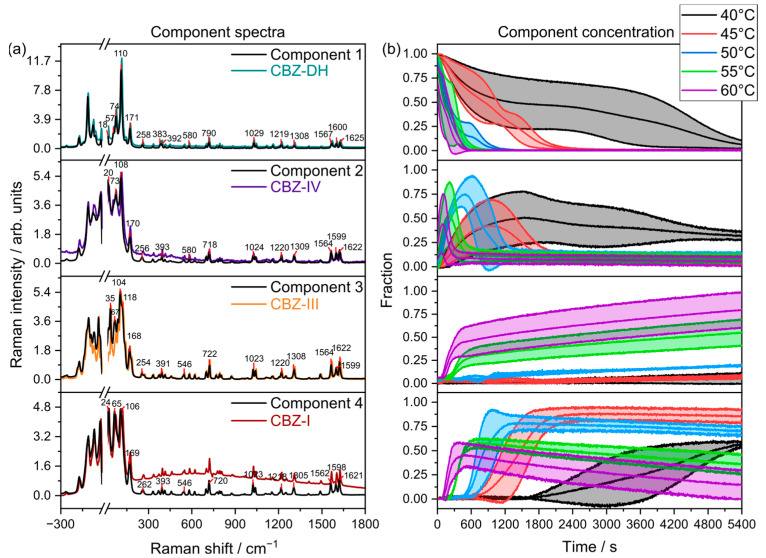
(**a**) LFR+MFR component spectra overlaid with their corresponding reference spectra (colored lines) collected at 20 °C (CBZ-DH, CBZ-DH, CBZ-IV, and CBZ-I). (**b**) Mean component concentrations between triplicate runs with standard deviations (shaded, n=3).

Multivariate curve resolution (MCR) is a type of unsupervised learning method which extracts pure response profiles of a species within unresolved mixtures. Unlike PCA, MCR analysis is guided by implied constraints. Therefore, MCR analysis was further used to semi-quantitatively elucidate the phase transformations of CBZ-DH under the explored temperature conditions. Pure components and their corresponding concentration profiles were calculated using all the replicate data from all five temperatures combined, as shown in Figure 5, Figure 6 and Figure 7. From the MCR analysis, it can be concluded that the behavior of CBZ-DH during its dehydration is temperature-dependent, with distinct solid-state transformation pathways observed at each temperature studied. In addition, according to the component concentration in Figure 5 and Figure 6, LFR and MFR spectral data indicate different CBZ-DH dehydration behavior. 

The MCR models using LFR spectral data (Figure 5) may be interpreted so that CBZ-DH (Component 1) transforms into three anhydrous polymorphs—CBZ-I (Component 4), CBZ-III (Component 3), and CBZ-IV (Component 2)—during its dehydration with complex dynamics among the components. At lower temperatures, CBZ-DH dehydrates over a long period of time (about 4800 s at 40 °C and about 2400 s at 45 °C), and CBZ-IV is evident for prolonged periods. At these lower temperatures, CBZ-I is the major product, and there is little evidence for CBZ-III. As the temperature is increased, the dehydration becomes faster, and the presence of CBZ-IV diminishes, whereas CBZ-III becomes the dominant product, with CBZ-I initially growing and then depleting with time. 

Some caution is required in the interpretation of quantitative aspects of the MCR analysis. Although the MCR algorithm outputs “concentrations”—these are relative to each component. However, the fact that the component spectra closely match those of the actual polymorphs suggests that the fractional contributions are qualitatively meaningful with CBZ-IV being present in the dehydration experiments at relatively high (greater than half) fraction of the components. 

The MCR models developed using MFR spectral data (Figure 6) exhibited that CBZ-DH (Component 1) transforms into three anhydrous polymorphs—CBZ-I (Component 4), CBZ-III (Component 3), and CBZ-IV (Component 2)—during its dehydration. The MFR-based MCR models (Figure 6) displayed concentration profiles that showed similarities and differences from those observed after using the LFR-based MCR models (Figure 5). Similarities between the two MCR models include the following: (1) the dehydration of CBZ-DH resulted in a four-component system comprising CBZ-DH, CBZ-I, CBZ-III, and CBZ-IV; (2) faster growth of CBZ-III with increasing temperature; (3) gradual decrease of CBZ-I, becoming more rapid as the temperatures increased. Differences the MFR-based MCR models displayed included the following: (1) CBZ-III was the dominant solid-state product for all temperatures; (2) only a gradual increase of CBZ-IV was observed for all temperatures. In these fittings, it is important to note the relatively poor spectral agreement between the components and the reference data for each of the polymorphs. Specifically, CBZ-IV showed quite poor correspondence with the component spectra in the MFR region.

The MCR models developed using combined (LFR+MFR) spectral data (Figure 7) exhibited that, during its dehydration, CBZ-DH (Component 1) transforms into three anhydrous polymorphs: CBZ-I (Component 4), CBZ-III (Component 3), and CBZ-IV (Component 2).

The LFR+MFR-based MCR models displayed results similar to the LFR-based MCR models, where a four-component system occurred during the dehydration of CBZ-DH. The combination of LFR and MFR spectral regions suggests that the MFR region may have not captured certain processes as effectively as the LFR domain, highlighting the potential benefits LFR spectroscopy can provide for investigating transient solid-state behavior.

## 4. Conclusions

Carbamazepine displays a complex solid-state landscape with its five known anhydrous forms (CBZ-I, CBZ-II, CBZ-III, CBZ-IV, and CBZ-V) and one hydrate form (CBZ-DH). Each solid-state form has different hydrogen bonding patterns, and these solid-state forms were characterized using their unique low- and mid-frequency Raman spectra, which were elucidated experimentally and theoretically using periodic boundary DFT calculations with good correlation (MAD < 10 cm^−1^). The dehydration behavior of powdered CBZ-DH was individually described by Raman spectroscopy in the low- and mid-frequency regions. Both LFR and MFR regions showed that the dehydration of CBZ-DH involves a transformation into a mixture of multiple anhydrous CBZ solid-state forms (CBZ-I, CBZ-III, and CBZ-IV) of varying relative concentrations under different isothermal conditions (40 °C, 45 °C, 50 °C, 55 °C, and 60 °C). The LFR region described the growth kinetics of solid-state forms differently to the MFR region. The MFR region indicated that the dehydration of CBZ-DH resulted in mostly CBZ-III at all temperatures. In contrast, the LFR region indicated that the dehydration of CBZ-DH below 55 °C resulted in mostly CBZ-I, and the dehydration of CBZ-DH above 55 °C resulted in mostly CBZ-III. Therefore, intricate processes such as the rapid growth and decline of CBZ-IV were not efficiently observed by the MFR spectral region. This study highlighted the capability of LFR spectroscopy to observe intricate processes that may not be effectively captured by MFR spectroscopy. Further studies could explore the complementary use of LFR spectroscopy alongside differential scanning calorimetry and thermal gravimetric analysis for the accurate quantification of crystalline hydrates during heating. These results have implications for the implementation of LFR spectroscopy in the pharmaceutical manufacturing environment, along with complementary techniques such as thermogravimetric analysis, differential scanning calorimetry, X-ray diffraction, and optical microscopy, in order to provide comprehensive characterizations of pharmaceutical materials and manufacturing process monitoring.

## Data Availability

Data are contained within the article or Supplementary Material.

## Supplementary Materials

The following supporting information can be downloaded at: https://www.mdpi.com/article/10.3390/pharmaceutics15051526/s1, Figure S1: Hydrogen patterns of (a) CBZ-I, (b) CBZ-II, (c) CBZ-III, (d) CBZ-IV, (e) CBZ-V, and (f) CBZ-DH.; Figure S2: (a) Low- and (b) mid- frequency Raman spectra of attempted anhydrous carbamazepine form II (CBZ-II, blue) compared theoretical Raman spectra of CBZ-II (black).; Figure S3: Visualization of selected low-frequency vibrational modes of CBZ-I.; Figure S4: Visualization of selected low-frequency vibrational modes of CBZ-III.; Figure S5: Visualization of selected low-frequency vibrational modes of CBZ-IV.; Figure S6: Visualization of selected low-frequency vibrational modes of CBZ-DH.; Figure S7: Mid-frequency Raman (MFR) spectra of (a) CBZ-I, (b) CBZ-III, (c) CBZ-IV, and (d) CBZ-DH collected at 20 °C (red) and −190 °C (blue) in comparison to their corresposnding DFT calculated Raman spectra (black).; Figure S8: PCA loadings (left) and scores (right) for data collected from all temperatures and the mid-frequency Raman spectral region. For ease of visualization, the mean scores value at each time point for each replicate run is shown.; Figure S9. PCA loadings (left) and scores (right) for data collected from all temperatures and the combined low and mid-frequency Raman spectral region. For ease of visualization, the mean scores value at each time point for each replicate run is shown.; Table S1: Vibrational mode assignment for the calculated LFR spectrum of CBZ-DH. Bold assignments are considered as strong characteristic peaks.; Table S2: Vibrational mode assignment for the calculated LFR spectrum of CBZ-I. Bold assignments are considered as strong characteristic peaks.; Table S3: Vibrational mode assignment for the calculated LFR spectrum of CBZ-II. Bold assignments are considered as strong characteristic peaks.; Table S4: Vibrational mode assignment for the calculated LFR spectrum of CBZ-III. Bold assignments are considered as strong characteristic peaks.; Table S5: Vibrational mode assignment for the calculated LFR spectrum of CBZ-IV. Bold assignments are considered as strong characteristic peaks.; Table S6: Experimental and theoretical Raman peaks of CBZ-DH (medium to strong) and their mean average deviation.; Table S7: Experimental and theoretical Raman peaks of CBZ-I (medium to strong) and their mean average deviation.; Table S8: Experimental and theoretical Raman peaks of CBZ-III (medium to strong) and their mean average deviation.; Table S9: Experimental and theoretical Raman peaks of CBZ-IV (medium to strong) and their mean average deviation.; CRYSTAL output files for both geometry optimization and frequency calculations for CBZ-I, CBZ-II, CBZ-III, CBZ-IV, and CBZ-DH (ZIP).
