# Supplementary material for: Exploring the Solid-State Landscape of Carbamazepine during Dehydration: A Low Frequency Raman Spectroscopy Perspective
Source: Pharmaceutics. 2023 May 18;15(5):1526. doi: 10.3390/pharmaceutics15051526 (PMC10222055; doi:10.3390/pharmaceutics15051526)
Supplement: Supplementary file 1 [file pharmaceutics-15-01526-s001.zip › pharmaceutics-2372920-supplementary.pdf]

Table S1. Vibrational mode assignment for the calculated LFR spectrum of CBZ-DH. Bold assignments are considered as strong characteristic peaks.

Experimental Raman shift / cm^{-1}	Theoeretical Raman shift / cm^{-1}	Relative Intensity	Normal modes	Principal axis
**19**	**21**	**9**	**Translational**	**ac**
64	64	8	Torsional	abc
77	77	25	Torsional	ab
86	89	11	Torsional	ac
**115**	**119**	**100**	**Torsional**	**abc**
129	125	30	Torsional	abc
147	151	7	Torsional	abc
172	175	24	Torsional	abc
260	258	5	Torsional	abc

Table S2. Vibrational mode assignment for the calculated LFR spectrum of CBZ-I. Bold assignments are considered as strong characteristic peaks.

Experimental Raman shift / cm^{-1}	Theoeretical Raman shift / cm^{-1}	Relative Intensity	Normal modes	Principal axis
**24**	**23**	**100**	**Tranlational**	**ac**
36	32	68	Translational	ab
43	49	39	Torsional	abc
**68**	**67**	**73**	**Torsional**	**abc**
**73**	**76**	**73**	**Torsional**	**abc**
90	97	99	Torsional	ac
**110**	**117**	**46**	**Torsional**	**ac**
**122**	**127**	**52**	**Torsional**	**bc**
172	175	38	Torsional	abc
**265**	**267**	**5**	**Torsional**	**ab**

Table S3. Vibrational mode assignment for the calculated LFR spectrum of CBZ-II. Bold assignments are considered as strong characteristic peaks.

Theoeretical Raman shift / cm^{-1}	Relative Intensity	Normal modes	Principal axis
**21**	**100**	**Translational**	**ac**
**28**	**58**	**Torsional**	**abc**
**61**	**40**	**Torsional**	**abc**
**67**	**73**	**Torsional**	**ac**
87	52	Torsional	abc
**108**	**34**	**Torsional**	**abc**
118	36	Torsional	abc
126	26	Torsional	abc
144	8	Torsional	abc
**171**	**10**	**Torsional**	**ac**
250	1	Torsional	abc
**261**	**4**	**Torsional**	**abc**
273	2	Torsional	abc

Table S4. Vibrational mode assignment for the calculated LFR spectrum of CBZ-III. Bold assignments are considered as strong characteristic peaks.

Experimental Raman shift / cm^{-1}	Theoeretical Raman shift / cm^{-1}	Relative Intensity	Normal modes	Principal axis
**42**	**48**	**37**	**Translational**	**ac**
51	53	15	Torsional	abc
68	72	3	Torsional	abc
76	89	21	Torsional	ab
**92**	**97**	**40**	**Torsional**	**ab**
**105**	**110**	**24**	**Torsional**	**ab**
**118**	**124**	**32**	**Torsional**	**abc**
**130**	**139**	**55**	**Torsional**	**abc**
**144**	**149**	**64**	**Torsional**	**ab**
169	171	15	Torsional	abc
175	181	9	Torsional	ac
185	191	43	Torsional	ab
253	254	6	Torsional	ab
276	278	9	Torsional	ab

Table S5. Vibrational mode assignment for the calculated LFR spectrum of CBZ-IV. Bold assignments are considered as strong characteristic peaks.

Experimental Raman shift / cm^{-1}	Theoeretical Raman shift / cm^{-1}	Relative Intensity	Normal modes	Principal axis
**24**	**20**	**100**	**Translational**	**bc**
**51**	**58**	**18**	**Translational**	**ac**
**68**	**70**	**31**	**Translational**	**ab**
**75**	**84**	**49**	**Torsional**	**abc**
90	98	24	Torsional	abc
**115**	**127**	**79**	**Torsional**	**bc**
**172**	**189**	**22**	**Torsional**	**abc**
**255**	**252**	**5**	**Torsional**	**abc**
**262**	**262**	**2**	**Torsional**	**abc**

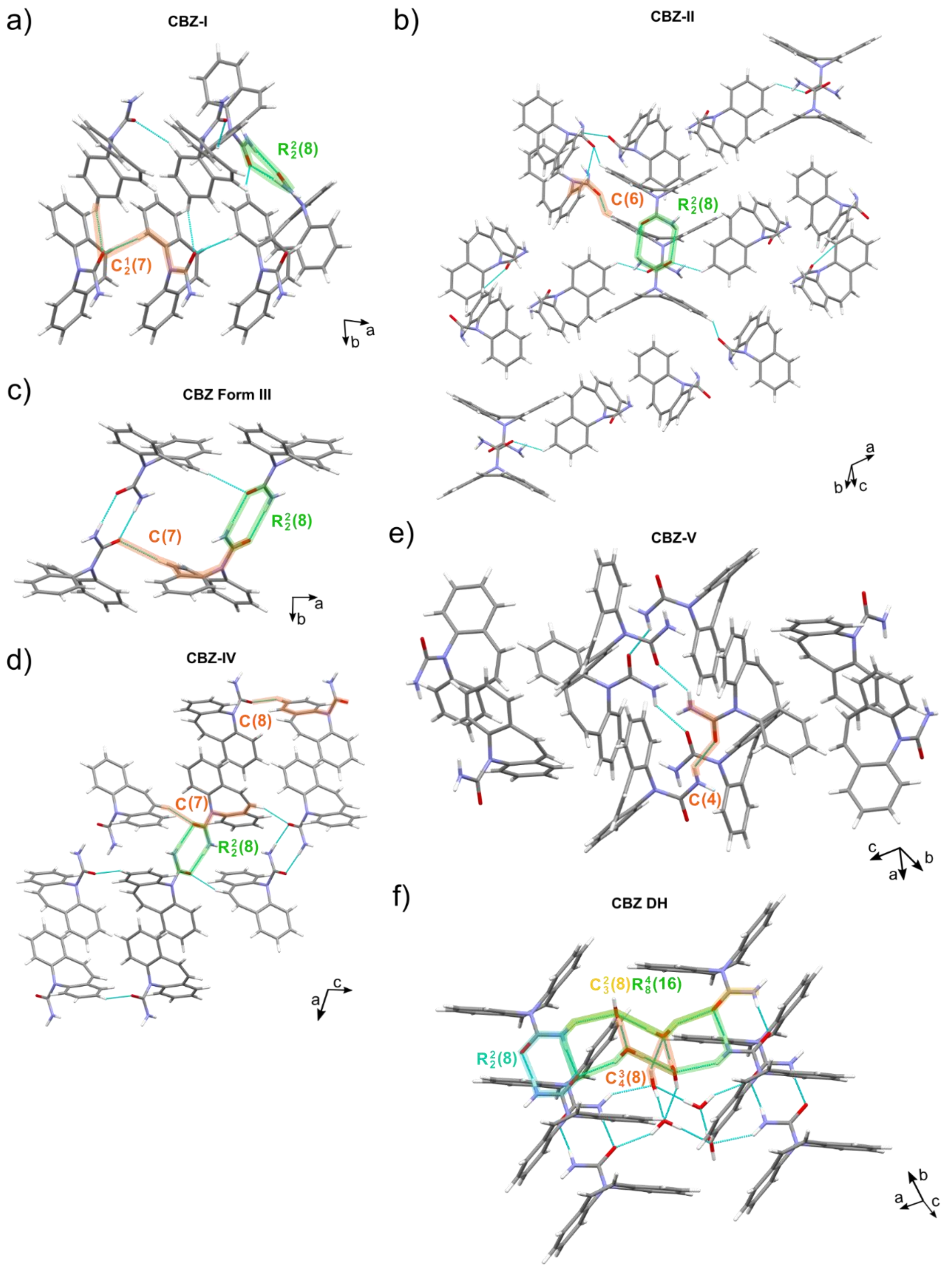

Figure S1. Hydrogen patterns of a) CBZ-I, b) CBZ-II, c) CBZ-III, d) CBZ-IV, e) CBZ-V, and f) CBZ-DH.

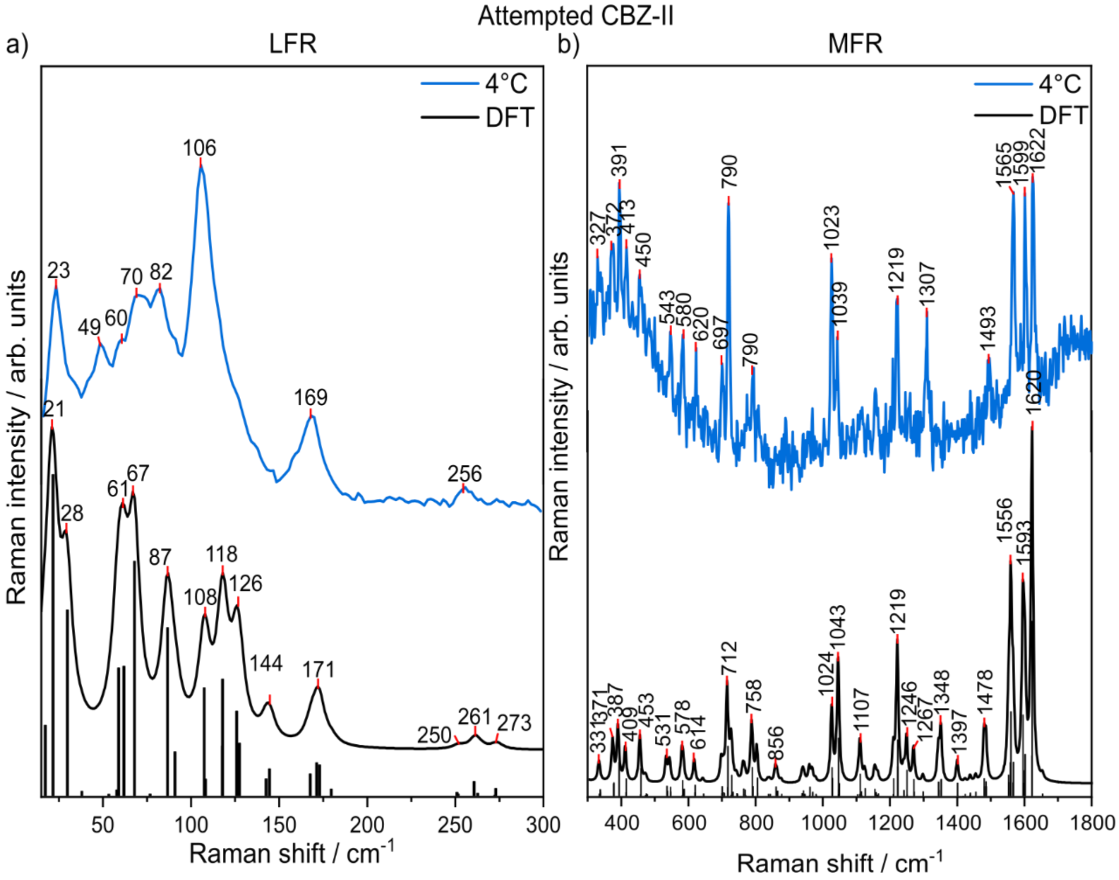

Figure S2. a) Low- and b) mid- frequency Raman spectra of attempted anhydrous carbamazepine form II (CBZ-II, blue) compared theoretical Raman spectra of CBZ-II (black).

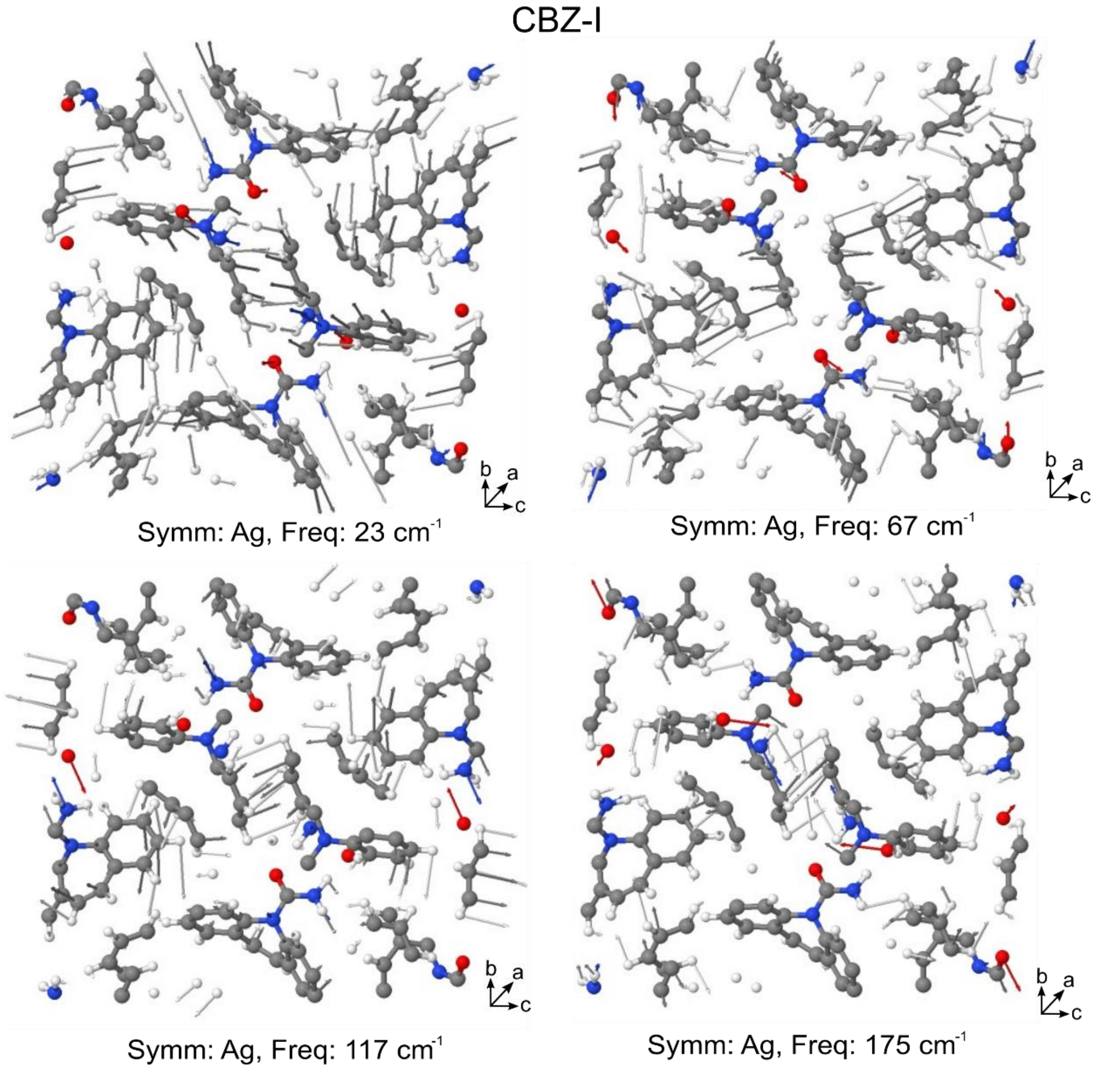

Figure S3. Visualization of selected low-frequency vibrational modes of CBZ-I.

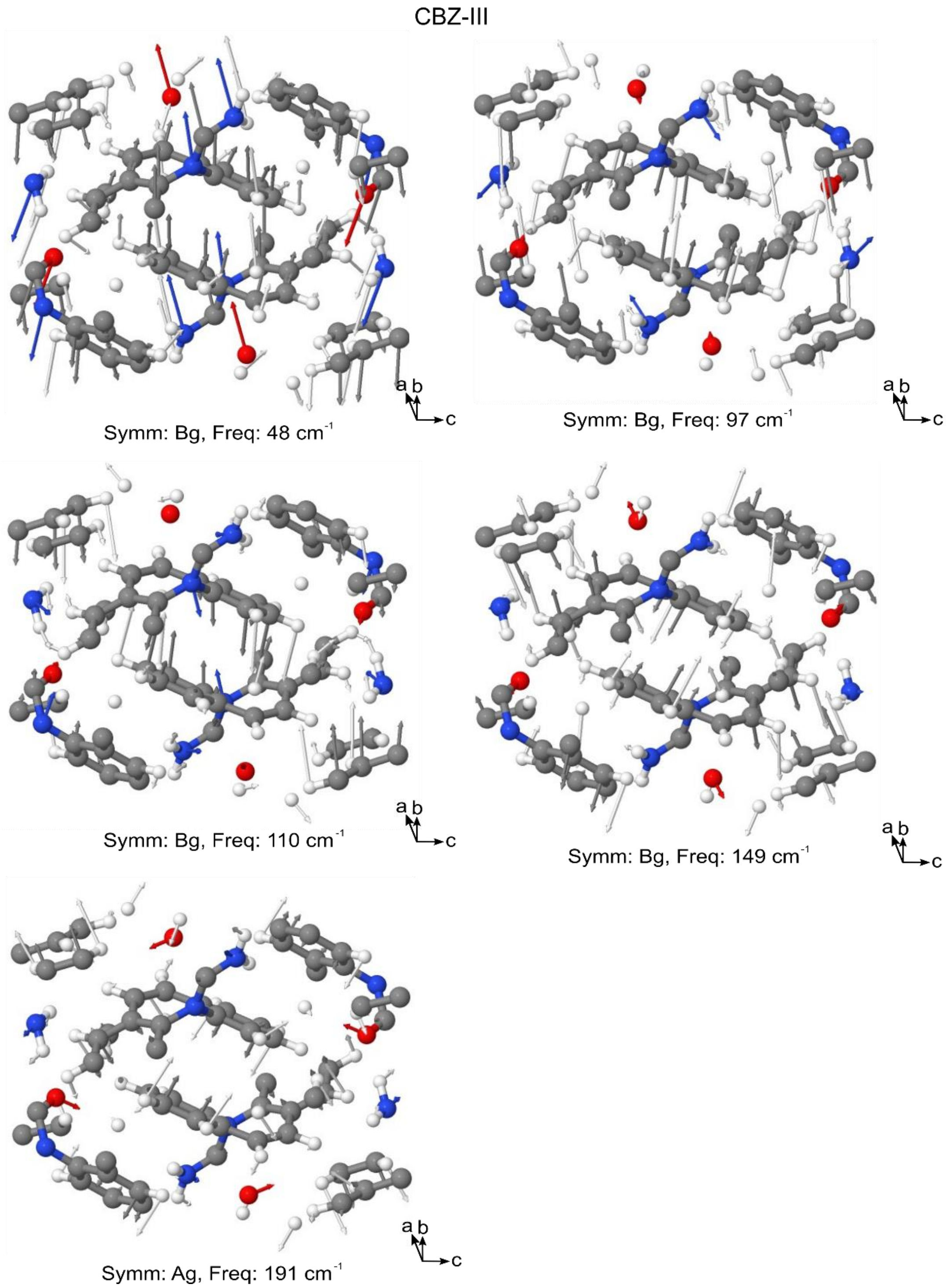

Figure S4. Visualization of selected low-frequency vibrational modes of CBZ-III.

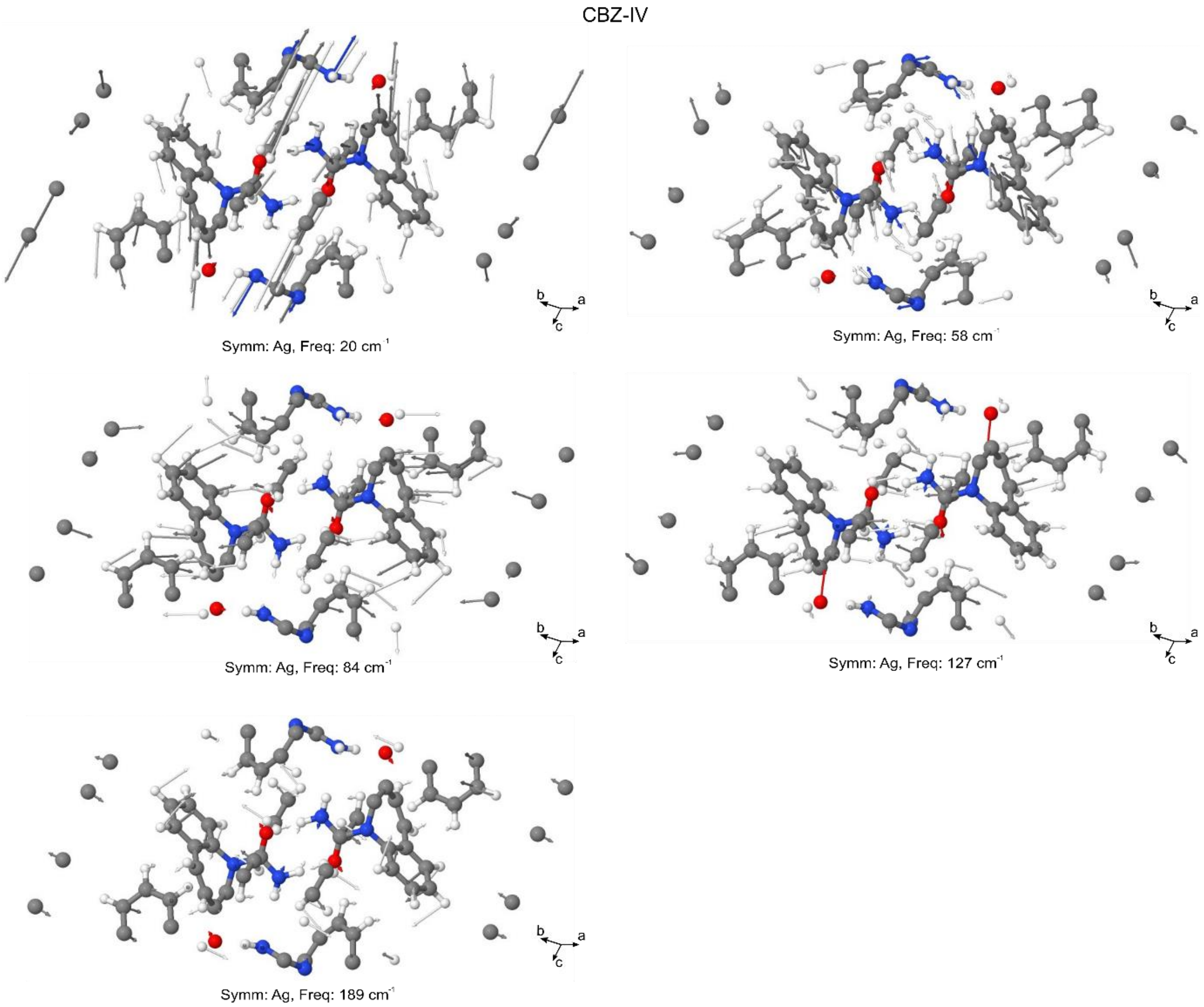

Figure S5. Visualization of selected low-frequency vibrational modes of CBZ-IV.

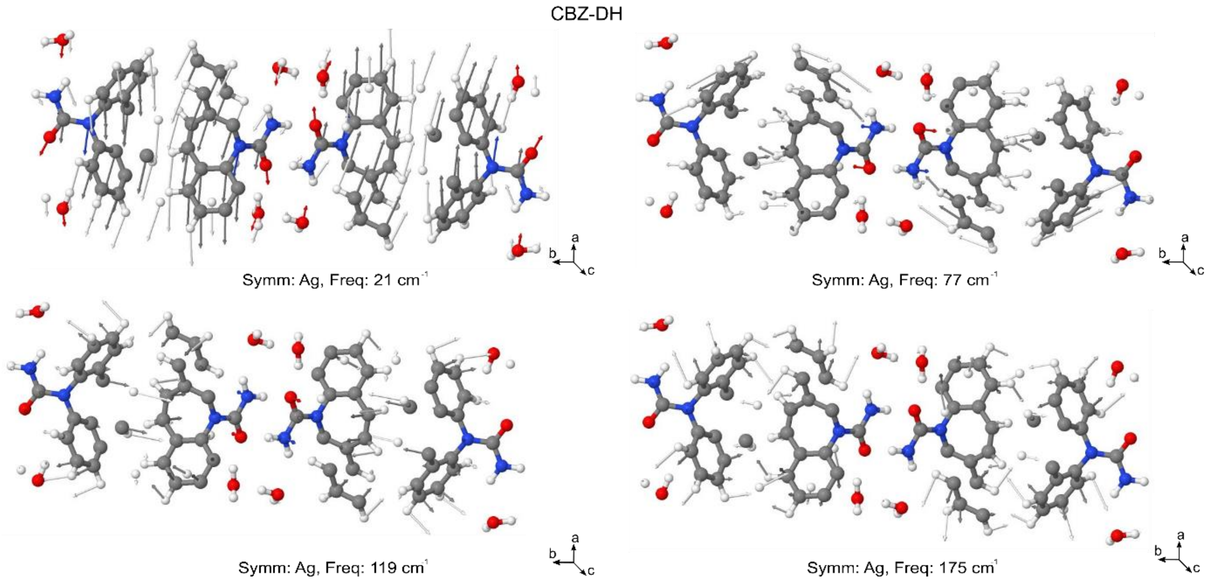

Figure S6. Visualization of selected low-frequency vibrational modes of CBZ-DH.

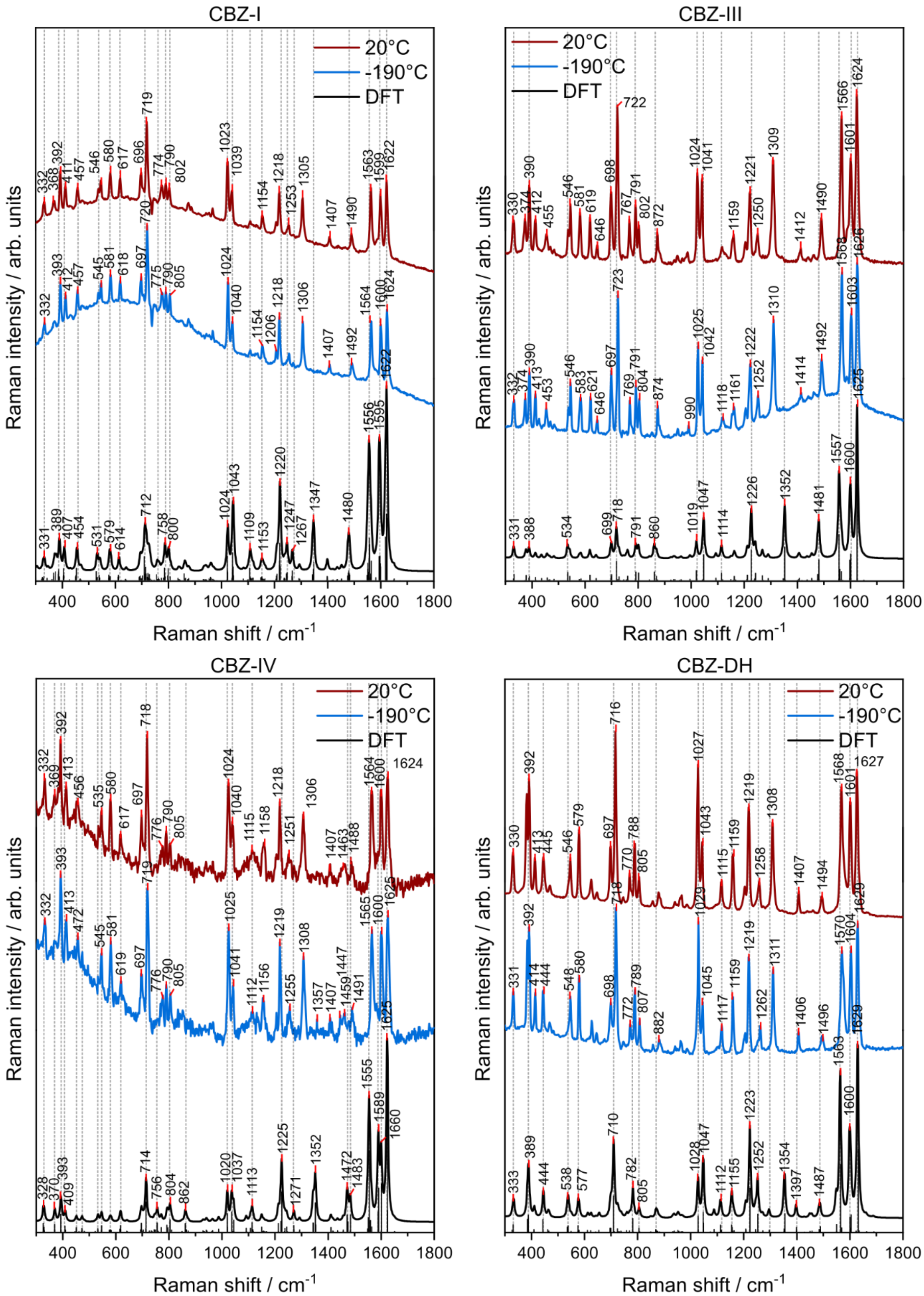

Figure S7. Mid-frequency Raman (MFR) spectra of a) CBZ-I, b) CBZ-III, c) CBZ-IV, and d) CBZ-DH collected at 20 °C (red) and -190 °C (blue) in comparison to their corresposnding DFT calculated Raman spectra (black).

Table S6. Experimental and theoretical Raman peaks of CBZ-DH (medium to strong) and their mean average deviation.

Experimental Raman shift / cm^{-1}	Theoeretical Raman shift / cm^{-1}	Mean average deviation / cm^{-1}
19	21	5.4
64	64	
77	77	
86	89	
115	119	
129	125	
147	151	
172	175	
260	258	
331	333	
392	389	
444	444	
548	538	
580	577	
718	710	
772	782	
807	805	
1029	1028	
1045	1047	
1117	1112	
1159	1155	
1219	1223	
1262	1252	
1311	1354	
1406	1397	
1496	1487	
1570	1563	
1604	1600	
1629	1629	

Table S7. Experimental and theoretical Raman peaks of CBZ-I (medium to strong) and their mean average deviation.

Experimental Raman shift / cm^{-1}	Theoeretical Raman shift / cm^{-1}	Mean average deviation / cm^{-1}
24	23	7.8
36	32	
43	49	
68	67	
73	76	
90	97	
110	117	
122	127	
172	175	
265	267	
332	331	
393	389	
412	407	
457	454	
545	531	
581	579	
618	614	
720	712	
775	758	
805	800	
1024	1024	
1040	1043	
1206	1153	
1218	1218	
1306	1347	
1492	1480	
1564	1556	
1600	1595	
1624	1622	

Table S8. Experimental and theoretical Raman peaks of CBZ-III (medium to strong) and their mean average deviation.

Experimental Raman shift / cm^{-1}	Theoeretical Raman shift / cm^{-1}	Mean average deviation / cm^{-1}
**42**	**48**	6.7
51	53	
68	72	
76	89	
**92**	**97**	
**105**	**110**	
**118**	**124**	
**130**	**139**	
**144**	**149**	
169	171	
175	181	
185	191	
253	254	
276	278	
332	331	
390	388	
546	534	
791	791	
874	860	
1025	1019	
1042	1047	
1118	1114	
1222	1226	
1310	1352	
1492	1481	
1568	1557	
1603	1600	
1626	1625	

Table S9. Experimental and theoretical Raman peaks of CBZ-IV (medium to strong) and their mean average deviation.

Experimental Raman shift / cm^{-1}	Theoeretical Raman shift / cm^{-1}	Mean average deviation / cm^{-1}
**24**	**20**	6.3
**51**	**58**	
**68**	**70**	
**75**	**84**	
90	98	
**115**	**127**	
**172**	**189**	
**255**	**252**	
**262**	**262**	
**332**	**328**	
393	393	
**413**	**409**	
719	714	
776	756	
805	804	
1025	1020	
1041	1037	
1112	1113	
1219	1225	
1255	1271	
1357	1352	
1459	1472	
1491	1483	
1565	1555	
1600	1600	
1625	1625	

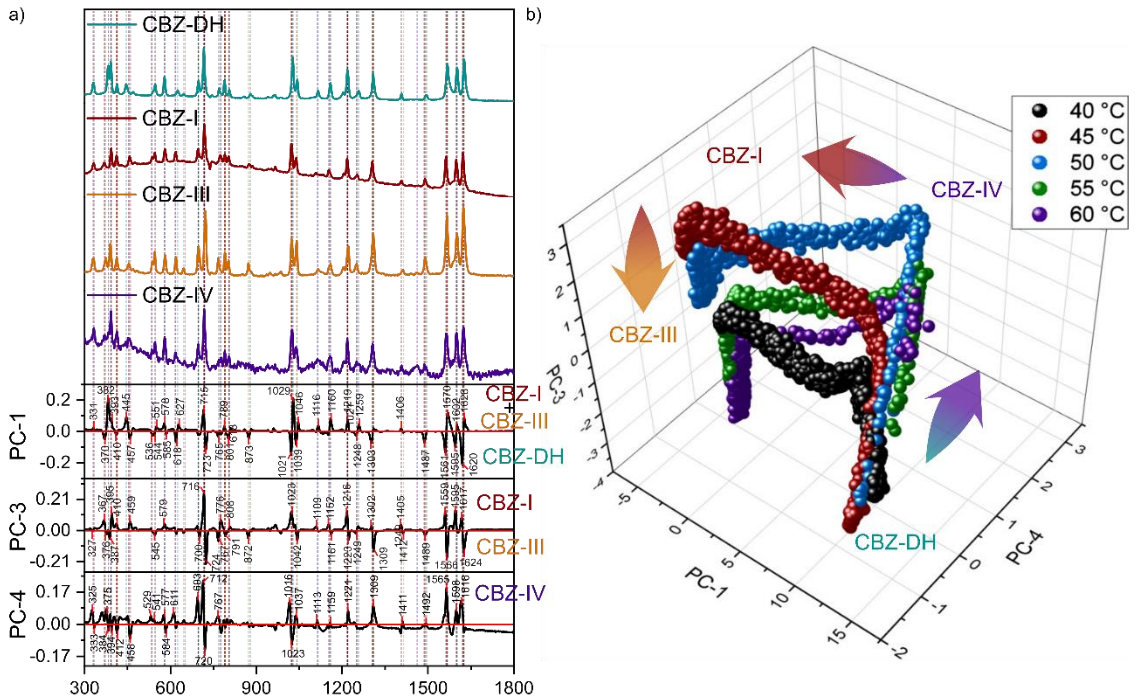

Figure S8. PCA loadings (left) and scores (right) for data collected from all temperatures and the mid-frequency Raman spectral region. For ease of visualization, the mean scores value at each time point for each replicate run is shown.

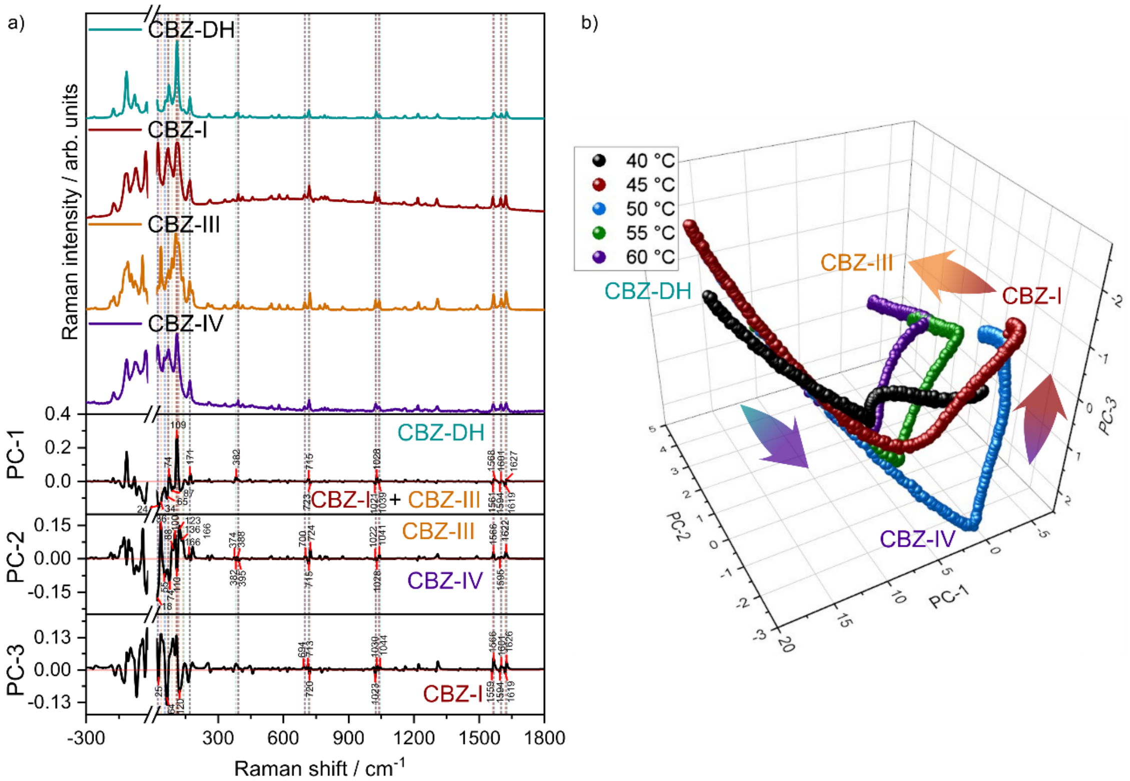

Figure S9. PCA loadings (left) and scores (right) for data collected from all temperatures and the combined low and mid-frequency Raman spectral region. For ease of visualization, the mean scores value at each time point for each replicate run is shown.
